# High-quality draft genome sequences of *Pseudomonas monteilii* DSM 14164^T^, *Pseudomonas mosselii* DSM 17497^T^, *Pseudomonas plecoglossicida* DSM 15088^T^, *Pseudomonas taiwanensis* DSM 21245^T^ and *Pseudomonas vranovensis* DSM 16006^T^: taxonomic considerations

**DOI:** 10.1099/acmi.0.000067

**Published:** 2019-10-29

**Authors:** Arantxa Peña, Antonio Busquets, Margarita Gomila, Magdalena Mulet, Rosa M. Gomila, Elena Garcia-Valdes, T. B. K. Reddy, Marcel Huntemann, Neha Varghese, Natalia Ivanova, I-Min Chen, Markus Göker, Tanja Woyke, Hans-Peter Klenk, Nikos Kyrpides, Jorge Lalucat

**Affiliations:** ^1^​ Department of Biology-Microbiology, Universitat de les Illes Balears, Palma de, Mallorca, Spain; ^2^​ Serveis Cientifico-Tècnics, Universitat de les Illes Balears, Palma de Mallorca, Spain; ^3^​ Institut Mediterrani d’Estudis Avançats (IMEDEA, CSIC-UIB), Palma de Mallorca, Spain; ^4^​ DOE Joint Genome Institute, 2800 Mitchell Drive, Walnut Creek, CA 94598-1698, USA; ^5^​ Leibniz Institute DSMZ - German Collection of Microorganisms and Cell Cultures, 38124 Braunschweig, Germany; ^6^​ School of Natural and Environmental Sciences, Newcastle University, Newcastle upon Tyne, NE1 7RU, UK

**Keywords:** Genomic Encyclopedia of Bacteria and Archaea (GEBA), Thousand Microbial Genomes Project (KMG I), *P. monteilii*, *P. mosselii*, *P. plecoglossicida*, *P. taiwanensis*, *P. vranovensis*, type strains, taxonomy

## Abstract

*
Pseudomonas* is the bacterial genus of Gram-negative bacteria with the highest number of recognized species. It is divided phylogenetically into three lineages and at least 11 groups of species. The *Pseudomonas putida* group of species is one of the most versatile and best studied. It comprises 15 species with validly published names. As a part of the Genomic Encyclopedia of Bacteria and Archaea (GEBA) project, we present the genome sequences of the type strains of five species included in this group: *Pseudomonas monteilii* (DSM 14164^T^), *Pseudomonas mosselii* (DSM 17497^T^), *Pseudomonas plecoglossicida* (DSM 15088^T^)*, Pseudomonas taiwanensis* (DSM 21245^T^) and *Pseudomonas vranovensis* (DSM 16006^T^). These strains represent species of environmental and also of clinical interest due to their pathogenic properties against humans and animals. Some strains of these species promote plant growth or act as plant pathogens. Their genome sizes are among the largest in the group, ranging from 5.3 to 6.3 Mbp. In addition, the genome sequences of the type strains in the *Pseudomonas* taxonomy were analysed via genome-wide taxonomic comparisons of ANIb, gANI and GGDC values among 130 *Pseudomonas* strains classified within the group. The results demonstrate that at least 36 genomic species can be delineated within the *P. putida* phylogenetic group of species.

## Introduction

The genus *Pseudomonas* is phylogenetically divided into three lineages and at least 19 groups and subgroups of species [1]. One of the most relevant is the *Pseudomonas putida* phylogenetic group of species, frequently called the *P. putida* species complex, because it is monophyletic and comprises 15 phenotypically closely related species [1–3]. Strains of species in this group are metabolically versatile and have attracted attention for their capability to use many different organic compounds, more recently for the presence of some species in clinical specimens [4, 5] and for their pathogenicity against animals (humans, fishes, insects) or plants. This group of species occupies many different ecological niches, such as soil, rhizosphere and water, as well as colonizing plants and animals [6]. The role of clinical and environmental strains of the *P. putida* phylogenetic group of species in the transfer of antimicrobial resistances has been recently studied by Peter *et al*. [7]. The difficulty in phenotypically identifying *Pseudomonas* species in clinical laboratories has been highlighted by Rebolledo and collaborators [8] and recently by Mulet and collaborators [9] for *P. putida* strains.

Previous publications based on three- or four-gene multilocus sequence analyses (MLSA) and phylogenomic analyses have demonstrated that the 15 recognized species within the *P. putida* phylogenetic group are monophyletic with high bootstrap support values at the branching nodes [2, 3, 10]. A phylogenetic tree based on the almost complete sequence of the 16S rRNA gene of the 15 type strains in the *P. putida* phylogenetic group, as well as a tree based on the concatenated sequences of the 16S rRNA, *gyrB* and *rpoD* partial gene sequences, has been published by Peña *et al*. [10]. ‘* Pseudomonas hunanensis *’ NCCB 100446 was also added to the present analysis because it was proposed as the type strain of a novel species [11], although its name has not yet been validly published under the rules of the International Code of Nomenclature of Bacteria (Bacteriological Code).

Comparative genomics is a very precise tool for species differentiation that can help in elucidating the taxonomy within the *P. putida* group of species; therefore, the whole-genome sequences of all the type strains in the group are needed. With this aim and in the frame of the GEBA project (Genomic Encyclopedia of Bacteria and Archaea, One Thousand Microbial Genomes Project=KMG I) [12, 13], we recently published the genome sequences of the *Pseudomonas fulva *, *Pseudomonas parafulva* and *Pseudomonas cremoricolorata* type strains [10]. In the present publication, we report the genome sequences of five additional type strains of species within the group: *Pseudomonas monteilii *, *Pseudomonas mosselii *, *Pseudomonas plecoglossicida, Pseudomonas taiwanensis* and *Pseudomonas vranovensis *. The first two strains have been isolated from clinical specimens, the third is a fish pathogen, and the last two are soil inhabitants [4, 5, 14–16]. We also discuss the affiliations of 130 strains that have been assigned taxonomically to *P. putida* or to other species within the group and whose complete genomes are publicly available. The type strains of the 15 recognized species within this group of species have been included in the analyses.

## Methods

### Organisms

The type strains of *P. monteilii* (DSM 14164^T^), *P. mosselii* (DSM 17497^T^), *P. plecoglossicida* (DSM 15088^T^)*, P. taiwanensis* (DSM 21245^T^) and *P. vranovensis* (DSM 16006^T^) have been provided by DSMZ. *P. monteilii* DSM 14164^T^ was isolated by Elomari *et al*. [5] from a human bronchial aspirate and was proposed as a representative type strain of a set of ten strains of the same species isolated from clinical specimens. Daboussi and collaborators studied 12 clinical strains received as *Pseudomonas fluorescens *, *P. putida* or *Pseudomonas* sp. that clustered together in a phenotypic numerical taxonomy study. The authors concluded in a polyphasic study that they represented a new species and proposed strain DSM 17497^T^ as the type strain of *P. mosselii* [4]. In a taxonomic study of four fish-pathogenic bacteria associated with haemorrhagic ascites of ayu (*Plecoglossus altivelis*), Nishimori *et al*. [14] proposed the new species *P. plecoglossicida* with strain DSM 15088^T^ as the type. *P. taiwanensis* DSM 21245^T^ [15] was isolated in a screening for chitin-degrading bacteria in soil samples, and *P. vranovensis* DSM 16006^T^ [16] was also isolated as a nitroaromatic compound degrader from a soil sample.

### Growth conditions and genomic DNA preparation

Strains were cultured aerobically in Luria–Bertani broth, with shaking at 30 °C, to the early stationary phase. Genomic DNA was extracted and purified with a Promega Wizard Genomic DNA Purification kit, following the manufacturer’s instructions. DNA quality and quantity were determined with a Nanodrop spectrometer (Thermo Scientific, Wilmington, USA).

### Genome sequencing and assembly

Methods have been previously published [10] and are summarized below. Draft sequencing, initial gap closure and annotation were performed by the DOE Joint Genome Institute (JGI) using state-of-the-art sequencing technology [17]. An Illumina standard shotgun library was constructed and sequenced using the Illumina HiSeq 2000 platform. Illumina sequencing and library artifacts were removed using Duk filtering (L. Mingkun, A. Copeland and H. J. Duk, unpublished data). Filtered Illumina reads were assembled using Velvet (version 1.1.04) [18], simulated paired-end reads were created from Velvet contigs using wgsim and simulated read pairs were reassembled using Allpaths-LG (version r42328) [19].

### Genome annotation

Protein-coding genes were identified using Prodigal [20] as part of the DOE-JGI genome annotation pipeline [21]. Additional gene prediction analysis and manual functional annotation were performed within the Integrated Microbial Genomes (IMG) platform, which provides tools for analysing and reviewing the structural and functional annotations of genomes in a comparative context [22]. Genome annotation procedures are detailed in Chen *et al*. [23] and references therein. Briefly, the predicted CDSs were translated and used to search the NCBI nonredundant database, UniProt, TIGRFAMs, Pfam, KEGG, COG and InterPro databases. Transfer RNA genes were identified using the tRNAscan-SE tool, and other noncoding RNAs were found using INFERNAL. Ribosomal RNA genes were predicted using hmmsearch against the custom models generated for each type of rRNA.

### Genome-wide comparative analysis

Whole-genome comparisons between pairs of genomes were calculated with three algorithms. Average nucleotide identity based on blast (ANIb) was calculated using the JSpecies software tool available at the webpage http://www.imedea.uib.es/jspecies [24, 25]. Genome-to-genome distance calculation (GGDC) was performed between genome pairs using the GGDC 2.0 update available via the web server http://ggdc.dsmz.de [26]. gANI was computed as pairwise bidirectional best nSimScan hits of genes having 70 % or more identity in at least 70 % coverage of the shorter gene [27]. The similarity matrix obtained with all pairwise genomic comparisons was used to generate a UPGMA dendrogram using the past software package version 3.20 [28].

### Chemotaxonomy: main protein profiles (MALDI-TOF MS)

Mass spectrometry analysis of whole cells was performed as previously described with a Bruker Autoflex mass spectrometer [10].

## Results and discussion

### Genome sequences

The genome project information is depicted in Table 1, and the data have also been deposited in the Genomes on Line Database (GOLD) [29]. High-quality draft genome sequences were obtained at the DOE-JGI and are deposited in GenBank and in the Integrated Microbial Genomes database (IMG) [30]. The GenBank IDs are JHYV01000000 for *P. monteilii* DSM 14164^T^, JHYW01000000 for *P. mosselii* DSM 17497 ^T^, JHYX01000000 for *P. plecoglossicida* DSM 15088^T^ *,* AUEC01000000 for *P. taiwanensis* DSM 21245^T^ and AUED01000000 for *P. vranovensis* DSM 16006^T^.

**Table 1.** Project information and genome statistics for *P. monteilii* DSM 14164^T^, *P. mosselii* DSM 17497^T^, *P. plecoglossicida* DSM 15088^T^, *P. taiwanensis* DSM 21245^T^ and *P. vranovensis* DSM 16006^T^. Data obtained from the Integrated Microbial Genomes (IMG) system [23]

	* P. monteilii * DSM 14164^T^	* P. mosselii * DSM 17497^T^	* P. plecoglossicida * DSM 15088^T^	* P. taiwanensis * DSM 21245^T^	* P. vranovensis * DSM 16006^T^
Property	Term	Term	Term	Term	Term
Finishing quality	Permanent draft, High-quality draft	Draft, High-quality draft	Permanent draft, High-quality draft	Permanent draft, High-quality draft	Permanent draft, High-quality draft
Libraries used	Illumina Regular Fragment, 270 bp	Illumina Regular Fragment, 270 bp	Illumina Regular Fragment, 270 bp	Illumina Regular Fragment, 270 bp	Illumina Regular Fragment, 270 bp
Sequencing platforms	Illumina HiSeq 2000	Illumina HiSeq 2000	Illumina HiSeq 2000	lumina HiSeq 2000	lumina HiSeq 2000
Average input read coverage used for the assembly	300X	300X	300X	122.2X	122.2X
Sequencing depth coverage	353X	390X	433X	348X	489X
Genome size (bp)	6 310 792	6 262 860	5 349 493	5 416 882	5 703 346
G+C content (%)	61.50	64.00	63.00	61.90	61.50
DNA coding (bp)	5 646 648	5 610 495	4 791 738	4 843 312	5 162,587
DNA G+C (bp)	3 880 258	4 004 956	3 368 736	3 349 604	3,507,852
DNA scaffolds	86	56	59	68	37
Total genes	6107	5916	4976	5092	5366
Protein-coding genes	5953	5763	4847	4974	5242
RNA genes	154	153	129	118	124
Pseudo genes	0	0	0	0	0
Protein-coding genes with function prediction	4662	4511	3972	4053	4290
Protein-coding genes with COGs	4007	3960	3529	3670	3961
in internal clusters	1004	939	586	607	803
with Pfam domains	4918	4803	4225	4292	4619
with signal peptides	657	634	511	532	626
with transmembrane helices	1365	1311	1108	1126	1195
CRISPR repeats	1	1	0	1	0
Assemblers	vpAllpaths v. r46652	vpAllpaths v. r46652	vpAllpaths v. r46652	Unknown program v. before 2013-03-26	Unknown program v. before 2013-03-26
Gene-calling method	Prodigal 2.5	Prodigal 2.5	Prodigal 2.5	Prodigal 2.5	Prodigal 2.5
Locus tag	Q381	Q380	Q378	H620	H621
GenBank ID	JHYV01000000	JHYW01000000	JHYX01000000	AUEC01000000	AUED01000000
GenBank date of release	16 August 2015	05 May 2014	05 May 2014	12 December 2013	12 December 2013
GOLD ID	Gp0021953	Gp0039999	Gp0040000	Gp0021955	Gp0021956
BIOPROJECT	PRJNA221052	PRJNA221051	PRJNA221049	PRJNA188913	PRJNA188914

### Genome properties

The assemblies of the five genomes, their properties and statistics are summarized in Table 1, and the numbers of genes associated with general COG functional categories are shown in Table 2. The G+C percentages for each strain were 61.49, 63.95, 62.97, 61.84 and 61.51, respectively. The majority of protein-coding genes (76.58, 76.61, 80.04, 79.73 and 80.06 %) were assigned a putative function.

**Table 2.** Number of genes associated with general COG functional categories

Code	* P. monteilii* DSM 14164^T^		* P. mosselii* DSM 17497^T^		* P. plecoglossicida* DSM 14164^T^		* P. taiwanensis* DSM 21245^T^		* P. vranovensis* DSM 16006^T^		Description
	Value	%	Value	%	Value	%	Value	%	Value	%	
J	237	5.18	246	5.45	234	5.87	243	5.82	238	5.25	Translation, ribosomal structure and biogenesis
A	1	0.02	1	0.02	1	0.03	1	0.02	1	0.02	RNA processing and modification
K	420	9.18	398	8.82	336	8.43	365	8.74	415	9.15	Transcription
L	163	3.56	153	3.39	124	3.11	121	2.90	124	2.73	Replication, recombination and repair
B	2	0.04	3	0.07	3	0.08	4	0.10	3	0.07	Chromatin structure and dynamics
D	43	0.94	45	1.00	39	0.98	40	0.96	38	0.84	Cell cycle control, cell division, chromosome partitioning
V	99	2.16	112	2.48	79	1.98	84	2.01	103	2.27	Defense mechanisms
X	73	1.60	73	1.62	44	1.10	16	0.38	48	1.06	Mobilome: prophages, transposons
w	32	0.70	39	0.86	23	0.58	28	0.67	34	0.75	Extracellular structures
T	289	6.32	301	6.67	248	6.22	282	6.75	286	6.31	Signal transduction mechanisms
M	247	5.40	255	5.65	230	5.77	241	5.77	244	5.38	Cell wall/membrane biogenesis
N	130	2.84	137	3.04	139	3.49	134	3.21	119	2.62	Cell motility
U	114	2.49	139	3.08	101	2.53	73	1.75	86	1.90	Intracellular trafficking and secretion
O	165	3.61	179	3.97	155	3.89	160	3.83	157	3.46	Posttranslational modification, protein turnover, chaperones
C	298	6.51	283	6.27	263	6.60	273	6.53	298	6.57	Energy production and conversion
G	211	4.61	184	4.08	174	4.37	187	4.48	202	4.45	Carbohydrate transport and metabolism
E	497	10.86	455	10.08	427	10.72	486	11.63	475	10.47	Amino acid transport and metabolism
F	96	2.10	93	2.06	92	2.31	97	2.32	96	2.12	Nucleotide transport and metabolism
H	231	5.05	211	4.67	209	5.24	213	5.10	218	4.81	Coenzyme transport and metabolism
I	188	4.11	193	4.28	187	4.69	196	4.69	275	6.06	Lipid transport and metabolism
P	293	6.40	273	6.05	218	5.47	236	5.65	289	6.37	Inorganic ion transport and metabolism
Q	115	2.51	124	2.75	96	2.41	113	2.70	123	2.71	Secondary metabolites biosynthesis, transport and catabolism
R	391	8.54	366	8.11	328	8.23	362	8.66	398	8.77	General function prediction only
S	241	5.27	251	5.56	235	5.90	223	5.34	266	5.86	Function unknown
–	2100	34.39	1956	33.06	1447	29.08	1422	27.93	1405	26.18	Not in COGs

The total is based on the total number of protein-coding genes in the genome.

### Genome-wide comparative analysis by ANI and GGDC

**Fig. 1.**
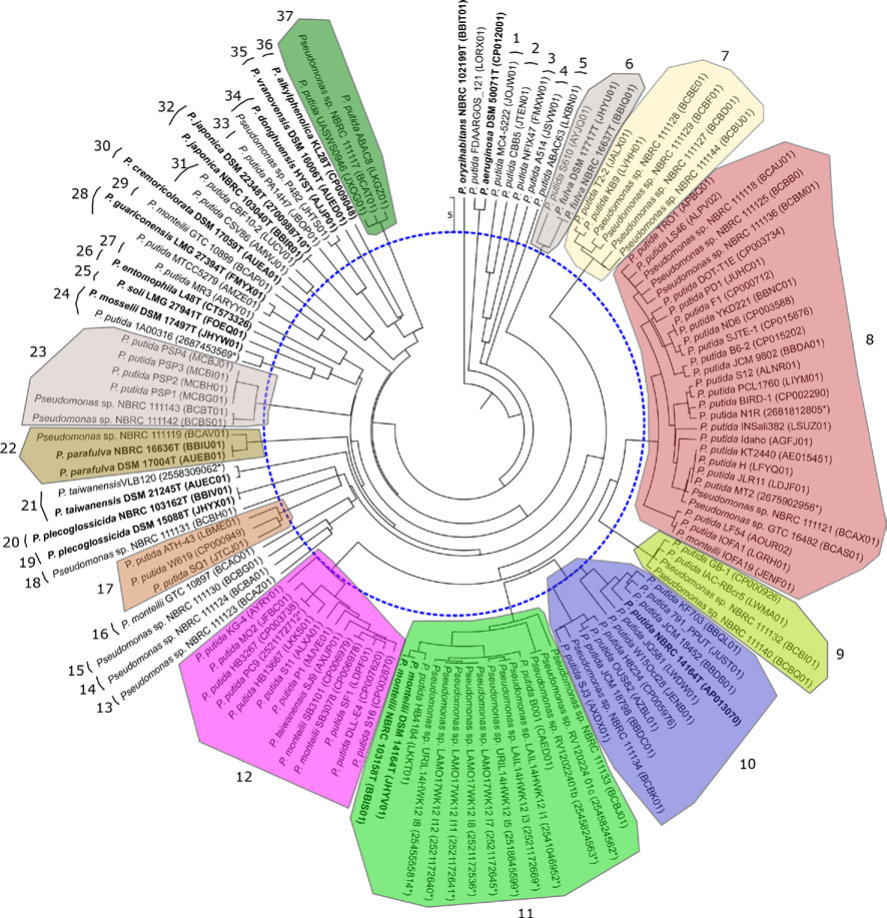
UPGMA dendrogram of the ANIb similarities of the genomes studied. The dotted line represents the recommended 95 % species threshold. Species type strains are labelled in bold. Groupings of strains using species cut-offs of 95 % are coloured and numbered.

**Fig. 2.**
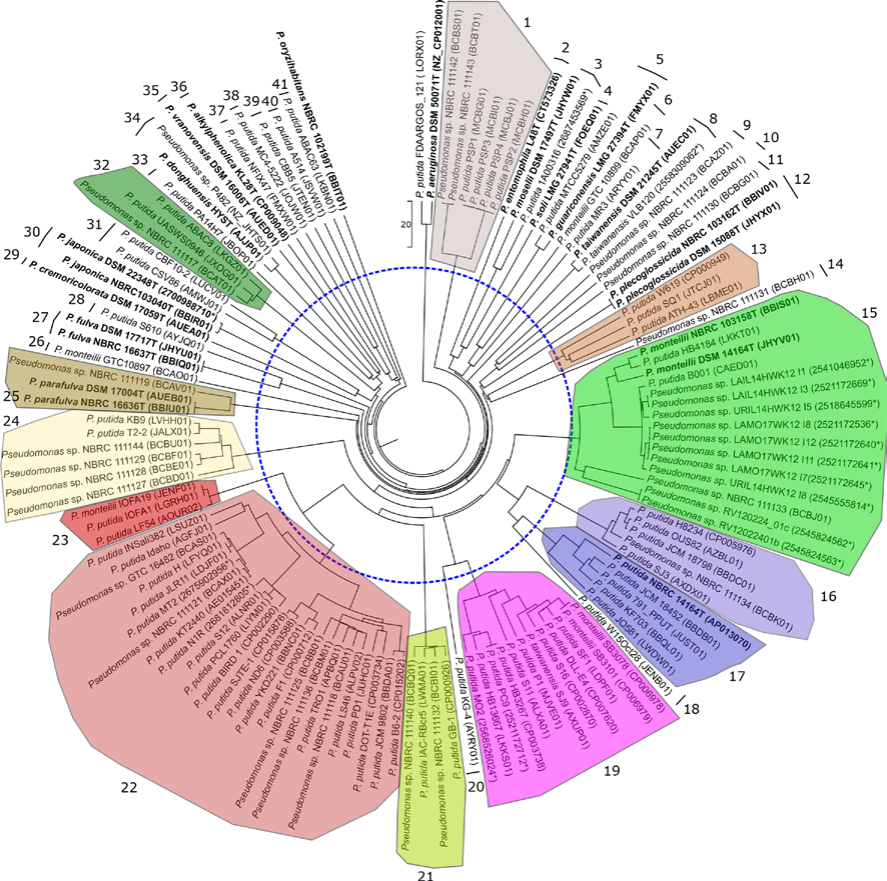
UPGMA dendrogram of the genome-to-genome distances of the genomes studied. The dotted line represents the recommended 70 % species threshold. Species type strains are labelled in bold. Groupings of strains using species cut-offs of 70 % are coloured and numbered.

Average nucleotide identity (ANI) is a very useful approach to calculate the similarities among bacterial strains and to cluster them in groups with ANI values higher than 95 % similarity, which correspond to genomic species. In the IMG website (https://img.jgi.doe.gov/cgi-bin/mer/main.cgi?section=ANI), the genome similarities among strains is computed by gANI, and clusters of strains with a similarity higher or equal to 95 % are considered a clique. In analysing the ANI values of these five type strains, we detected several discrepancies. For instance, the genomes of nine strains deposited as *P. monteilii* are distributed into five cliques; two genomes deposited for *P. mosselii* strains were allocated to clique 740 (together with *P. putida* 1A00316) and clique 7872 (* P. mosselii* strain SJ10, a singleton); five genomes of *P. plecoglossicida* strains were distributed in two cliques; two genomes of *P. taiwanensis* were distributed in two cliques; *P. vranovensis* was a singleton. Clique 2323 contains 13 strains: three assigned to *P. monteilii *, two to *P. plecoglossicida *, five to *P. putida *, one to *P. taiwanensis* and two were assigned to *Pseudomonas* spp. These observations prompted us to calculate the ANI values of 130 genomes deposited in public databases and assigned to species in the *P. putida* phylogenetic group. The strains analysed and accession numbers of their genomes are given in Table S1 (available in the online version of this article). The genomes of the 15 recognized species in the group were included. To ensure the taxonomic value of the ANI groupings, we also calculated two other indices: the GGDC values, an algorithm very useful for detecting clusters of strains that correspond to genomic species [26, 31] and a three-gene MLSA as previously described [32]. Figs 1 and 2 show the dendrograms for the ANIb and GGDC values. Both methods tested gave the same groupings of strains using species cut-offs of 70 % (GGDC), 95 % (ANIb and gANI) and 97 % (MLSA). Clear gaps were detected between the groupings. Good concordance was observed between the ANIb and GGDC groupings, as shown in Table S1, with a few exceptions: ANIb grouping 6 was split into two GGDC groupings (27, 28); ANIb grouping 8 into GGDC groupings 22 and 23; ANIb grouping 10 into GGDC groupings 16, 17 and 18; and ANIb grouping 12 into GGDC groupings 19 and 20. The mentioned GGDC groupings were on the threshold species delineation. Sixty strains whose genomes have been deposited did not cluster with the genome of the corresponding species type strain at the aforementioned species threshold levels. It is also surprising that 36 genomic species can be differentiated in the *P. putida* phylogenetic group, while only 15 are currently accepted with species status. For example, the 13 strains in the aforementioned clique 2323 clustered as a unique genomospecies 20 in the dendrogram, and no species type strain was in that cluster.

### Chemotaxonomy

Whole-cell protein profiles are very useful for the rapid identification of bacteria and are frequently used in clinical laboratories. Therefore, the major proteins were determined for the five type strains, and the mass to charge ratios of the proteins are given in Table S2. Protein profiles were compared with the other species type strains in the group [10], indicating that they can be used for identification and chemotaxonomic purposes as previously reported [2]. As shown in Fig. S1 the five type strains can be clearly differentiated in their protein profiles and were concordant with the corresponding profiles of the three type strains available in the Biotyper database.

### Comparative analysis of genes relevant for taxonomic characteristics and for species differentiation

Genes coding for general phenotypic properties of species in the genus *Pseudomonas* were detected in the five type strains: cell motility, chemotaxis and flagellar assembly. Related to the oxidative metabolism of *Pseudomonas *, the following genes encoding enzymes characteristic of aerobic microorganisms were detected: cytochrome coxidase, catalase and superoxide dismutase. All the strains have genes coding for catecholate siderophore receptors, which are considered phenotypic characteristics of the iron-chelating systems used for species differentiation in the genus *Pseudomonas* [33, 34]. The five species studied are considered strictly aerobic, and no denitrification genes have been found in their genomes. Only *P. plecoglossicida-* and *P. vranovensis *-type strains have been described as able to reduce nitrate. Both these strains have a gene coding for an assimilatory nitrate/nitrite reductase.

The utilization of arginine by *Proteobacteria* has attracted attention for many years and is a biochemical property routinely tested by taxonomists. The arginine deiminase (‘dihydrolase’) test is included in commercial kit systems. The arginine deiminase reactions consist of the conversion of arginine to citrulline and of citrulline to ornithine with liberation of ammonia. This system provides a limited energy gain under anaerobic conditions; it is inhibited under aerobic conditions and is encoded by *arcD* (arginine/ornithine antiporter), *arcA* (arginine deiminase), *arcB* (ornithine carbamoyltransferase) and *arcC* (carbamate kinase). In accordance with the species descriptions, all four genes are present in the five type strains studied and are organized in the same order.

Structural genes for type IV pilus synthesis, associated with twitching motility, were found in four of the type strains, but not in *P. taiwanensis *. The *P. monteilii* and *P. vranovensis* genomes code for additional conjugal transfer pilus proteins.

A high percentage of genes coding for proteins connected to KEGG pathways (27 to 31 %) allow us to infer other biochemical properties. Genes for key enzymes in the catabolism of carbohydrates demonstrate that hexoses are channelled to the glycolysis (phosphofructokinase), pentose phosphate and Entner–Doudoroff (2-keto-3-deoxy-phosphogluconate dehydrogenase) pathways in all type strains. Glucoamylase for starch degradation is also present in the five type strains, although in the original description *P. mosselii* and *P. vranovensis* were reported as starch negative [4, 16]. The ability to assimilate starch was not reported for the other three species.

Many *P. putida* and related strains have been studied for their biodegradative capabilities toward anthropogenic compounds and for their use in environmental biotechnology. The diversity of aromatic degradation pathways in the group is a good example of the metabolic potential of the five species considered. *P. mosselii* has genes coding for the degradation of 4-hydroxy-phenylacetate via protocatechuate by means of an extradiol aromatic ring-opening enzyme. Genes coding for salicylate and 3-methylsalicylate hydroxylases were found, but none were found for the catechol or 3-methylcatechol dioxygenases that are expected to continue the ortho or meta degradative catechol pathway. *P. monteilii* presents a complete set of genes for the catabolism of phenol, which includes the six subunits of phenol hydroxylase and the subsequent ortho or meta pathways by means of the catechol-1,2 or catechol 2,3-dioxygenases. *P. monteilii *, *P. plecoglossicida *, *P. taiwanensis* and *P. vranovensis* strains have genes for enzymes needed for benzoate catabolism through catechol-1,2-dioxygenase (ortho pathway) and for a protocatechuate-3,4-dioxygenase for p-hydroxybenzoate catabolism in *P. taiwanensis *.

Strains in the *P. putida* phylogenetic group of species have also been studied for their capacity to synthesize poly-beta-hydroxyalkanoate (PHA) granules, although *Pseudomonas* was initially considered unable to accumulate poly-beta-hydroxybutyrate (PHB) [6]. All five strains studied have a complete set of genes for the synthesis of PHA, including the polyhydroxyalkanoate synthase gene and the PHA granule-associated protein phasin. The ability to accumulate PHA as a reserve material has not been reported in the five original species proposals, but the five type strains were considered negative for the PHB production.

At least six different secretion systems (SS) have been described in species of the genus *Pseudomonas *. Type 1 and type 2 secretion systems (T1SS and T2SS) are considered general systems, but T3SS, T4SS and T6SS might be associated with virulence factors for plants and animals, and the presence of one of these three types of secretion systems in a strain is considered an indication of potential pathogenicity and is therefore important for risk assessment regarding the use of bacteria in bioaugmentation processes for bioremediation. The *P. monteilii *, *P. mosselii* and *P. plecoglossicida* type strains were isolated from clinical specimens or from haemorrhagic ascites and have genes for the synthesis of types 3 and 6 secretion systems, together with the secreted effectors HcP and VgrG. The *P. monteilii* and *P. mosselii* type strain genomes also contain genes for a T4SS. The *P. taiwanensis* and *P. vranovensis* strains are of environmental origin, and none has genes for a T3SS, but both possess the genes for a T6SS associated with the effectors HcP and VgrG. *P. taiwanensis* shows a remarkably high number of additional effectors, such as ImpK, ImpL, VarG and six copies of VgrG.

The agents that provide the transfer of genetic material among bacteria are distributed into four classes and generally classified in the genome as the mobilome [35]. Genes in this category include transposons, insertion elements, plasmids, bacteriophages and integrating chromosomal elements. The mobilome represents the adaptation of strains to changing environmental conditions; it is strain-specific in many instances and the main reason for bacterial intraspecies variability [36]. The four aforementioned mobile elements have been found in the studied type strains and are summarized in Table 3. Clusters of genes coding for phage proteins were found in the five strains: one cluster in *P. plecoglossicida* and *P. taiwanensis *, two in *P. monteilii *, three in *P. vranovensis* and five in *P. mosselii *. A remarkably high number of mobile genetic elements were found in the two clinical strains. *P. monteilii* has 237 genes distributed in three putative integrating chromosomal elements (ICE) and two mobile elements: ICE1, with 60 genes; ICE2, with 41 genes that include integration and transfer genes, as well as a toxin/antitoxin pair of genes; ICE3, with genes coding for transfer properties; mobile element 1, consisting of 20 genes, including Co, Zn and Cd resistance genes together with genes related to plasmids; and mobile element 2, containing 12 genes for the synthesis of conjugal transfer pili and conjugal transfer. The *P. mosselii* genome also contains five putative ICEs and mobile elements: ICE1 contains a ParD/ParE1 type toxin/antitoxin system and ICE2, with 76 genes, contains transfer genes and genes related to Hg, Co, Zn and Cd resistance. CRISPR arrays were found in all but *P. plecoglossicida *, and *P. vranovenis* type strains. The highest numbers of putative transposases, insertion elements and integrases were found in the pathogenic species *P. monteilii* (103), *P. mosselii* (71) and *P. plecoglossicida* (50).

**Table 3.** Genes included in the mobilome category for *P. monteilii* DSM 14164^T^, *P. mosselii* DSM 17497^T^, *P. plecoglossicida* DSM 15088^T^, *P. taiwanensis* DSM 21245^T^ and *P. vranovensis* DSM 16006^T^ Number of genes, their ID and their position in the chromosome are indicated for each type of mobile element detected.

	* P. monteilii * DSM 14164^T^	* P. mosselii * DSM 17497^T^	* P. plecoglossicida * DSM 15088^T^	* P. taiwanensis * DSM 21245^T^	* P. vranovensis * DSM 16006^T^
Type of mobile genes	Q381DRAFT_	Q380DRAFT_	Q378DRAFT_	H620DRAFT_	H621DRAFT_
Prophage 1	01819-01853 35 genes	01539-01552 14 genes	02943-02992 50 genes	04595-04663 69 genes	00220-00266 47 genes
Prophage 2	02854-028278 24 genes	03320-03375 56 genes			00490-00413 78 genes
Prophage 3		03884-03919 36 genes			01743-01765 23 genes
Prophage 4		04931-04946 16 genes			
Prophage 5		O5397-05413 17 genes			
ICE 1	0920-0979 60 genes	03803-03835 34 genes			
ICE 2	01690-1730 41 genes	04259-04325 67 genes			
ICE 3	04874-04969 34 genes	00238-00317 76 genes			
Mobile element 1	00610-00591 20 genes	05448-05482 35 genes			
Mobile element 2	05230-05241 12 genes				
CRISPR array count	2	1		1	
Transposases and insertion elements	86	61	42	13	22
Integrases	17	10	8	6	10

ICE: Integrating chromosomal element; CRISPR: clustered regularly interspaced short palindromic repeats.

### Conclusions

The genomic features observed here are in accordance with the characteristic metabolic versatility of *Pseudomonas* species and its capacity to occupy many environmental niches, including animal hosts, as evident by the pathogenic properties of some members of the group. The differentiation among pathogenic and nonpathogenic strains is of crucial importance, especially in the clinical laboratory. The large discrepancies detected in the comparison of genomes by ANI and GGDC demonstrate the need for a thorough revision of the taxonomy of strains assigned to species in the *P. putida* phylogenetic group. Accepting the predominant value of genome characteristics for the delineation of species in the actual taxonomy, we conclude that at least 36 genomic species can be delineated within the *P. putida* phylogenetic group of species, while only 15 are currently accepted with species status. Deeper phylogenomic and taxonomic analyses are needed to justify the proposal of 21 new species within the genus. Knowledge of the genome features of all bacterial species type strains is crucial to construct a reliable and stable bacterial taxonomy.

## Supplementary Data

Supplementary material 1
