# Supplementary material for: High-quality draft genome sequences of Pseudomonas monteilii DSM 14164T, Pseudomonas mosselii DSM 17497T, Pseudomonas plecoglossicida DSM 15088T, Pseudomonas taiwanensis DSM 21245T and Pseudomonas vranovensis DSM 16006T: taxonomic considerations
Source: Access Microbiol. 2019 Oct 29;1(10):e000067. doi: 10.1099/acmi.0.000067 (PMC7491935; doi:10.1099/acmi.0.000067)
Supplement: Supplementary material 1 [file acmi-1-067-s001.pdf]

Supplementary material

Table S1. Strains included in the study, genome characteristics, accession numbers of their genomes and groupings in the ANIb and GGDC analyses.

Table S2. Major protein profiles of *P. monteilii* DSM 14164^T, *P. mosselii* DSM 17497^T, *P. plecoglossicida* DSM 15088^T, *P. taiwanensis* DSM 21245^T and *P. vranovensis* DSM16006^T (m/z: mass to charge ratio; + and -: presence or absence of the corresponding protein).

Figure S1. Dendrogram based on the comparison with the Biotyper program of the major proteins detected. The profiles of closely related type strains present in the Biotyper database are also included as a control and are labeled with an asterisk. Distances generated are arbitrary.

Supplementary Table S1. Strains included in the study, genome characteristics, accession numbers of their genomes and groupings in the ANIb and GGDC analyses. Type strains are indicated in bold.

Species	Strain	Genome accession number	Size	Contigs	GC [%]	Genomic Group (ANIb)	Genomic Group (GGDC)
Pseudomonas alkylphenolica	**KL28**^{T}	**CP009048**	**5764622**	**1**	**60,6**	**35**	**36**
Pseudomonas cremoricolorata	**DSM 17059**^{T}	**AUEA01**	**4655082**	**26**	**63,5**	**29**	**29**
Pseudomonas donghuensis	**HYS**^{T}	**AJJP01**	**5639475**	**231**	**62,4**	33	**34**
Pseudomonas entomophila	**L48**^{T}	**CT573326**	**5888780**	**1**	**64,2**	**25**	**2**
Pseudomonas fulva	**NBRC 16637**^{T}	**BBIQ01**	**4768229**	**46**	**61,8**	**6**	**27**
Pseudomonas fulva	**DSM 17717**^{T}	**JHYU01**	**4770636**	**48**	**61,7**	**6**	**27**
Pseudomonas guariconensis	**LMG 27394**^{T}	**FMYX01**	**5079034**	**29**	**62,6**	**27**	**5**
Pseudomonas japonica	**NBRC 103040**^{T}	**BBIR01**	**6663130**	**162**	**64,2**	**31**	**30**
Pseudomonas japonica	**DSM22348**^{T}	**2700988710***	**6718608**	**61**	**64,1**	**31**	**30**
Pseudomonas monteilii	**DSM 14164**^{T}	**JHYV01**	**6308713**	**85**	**61,5**	**11**	**15**
Pseudomonas monteilii	GTC 10897	BCAO01	5547282	149	60,4	**16**	26
Pseudomonas monteilii	GTC 10899	BCAP01	5876827	112	62	28	6
Pseudomonas monteilii	IOFA19	JENF01	5725190	126	61,3	8	23
Pseudomonas monteilii	**NBRC 103158**^{T}	**BBIS01**	**6299985**	**132**	**61,5**	**11**	**15**
Pseudomonas monteilii	SB3078	CP006978	6000087	1	62,5	12	19
Pseudomonas monteilii	SB3101	CP006979	5945120	1	62,5	12	19
Pseudomonas mosselii	**DSM 17497**^{T}	**JHYW01**	**6260844**	**55**	**64**	**23**	**3**
Pseudomonas oryzihabitans	**NBRC 102199**^{T}	**BBIT01**	**5027590**	**34**	**66,2**	outgroup	
Pseudomonas parafulva	**NBRC 16636**^{T}	**BBIU01**	**4954362**	**55**	**62,5**	**21**	**25**
Pseudomonas parafulva	**DSM 17004**^{T}	**AUEB01**	**4956622**	**32**	**62,4**	**21**	**25**
Pseudomonas plecoglossicida	**DSM 15088**^{T}	**JHYX01**	**5347571**	**58**	**63**	**19**	**12**
Pseudomonas plecoglossicida	**NBRC 103162**^{T}	BBIV01	5341796	97	63	19	12
Pseudomonas putida	1A00316	2687453569*	5636969	43	64,5	23	3

Pseudomonas putida	791_PPUT	JUST01	6242976	576	62	10	17
Pseudomonas putida	A514	JSVW01	6767427	49	57,7	4	40
Pseudomonas putida	ABAC63	LKBN01	5139911	3451	62,3	5	41
Pseudomonas putida	ABAC8	LKGZ01	5543802	66	64,4	36	32
Pseudomonas putida	ATH-43	LBME01	5830220	64	61	17	13
Pseudomonas putida	B001	CAED01	5740939	262	62,2	11	15
Pseudomonas putida	B6-2	CP015202	6239598	27	61,6	8	22
Pseudomonas putida	BIRD-1	CP002290	5731541	1	61,7	8	22
Pseudomonas putida	CBB5	JTEN01	6817062	146	60	2	39
Pseudomonas putida	CBF10-2	LUCV01	6120625	73	63,7	30	31
Pseudomonas putida	CSV86	AMWJ01	6469780	209	63,1	30	31
Pseudomonas putida	DLL-E4	CP007620	6484062	1	62,5	12	19
Pseudomonas putida	DOT-T1E	CP003734	6260702	1	61,4	8	22
Pseudomonas putida	F1	CP000712	5959964	1	61,9	8	22
Pseudomonas putida	FDAARGOS_121	LORX01	6958027	3	66	outgroup	
Pseudomonas putida	GB-1	CP000926	6078430	1	61,9	9	21
Pseudomonas putida	H	LFYQ01	6059900	55	61,6	8	22
Pseudomonas putida	H8234	CP005976	6870827	1	61,6	10	16
Pseudomonas putida	HB13667	LKKS01	6325504	150	62,4	12	19
Pseudomonas putida	HB3267	CP003738	5875750	1	62,6	12	19
Pseudomonas putida	HB4184	LKKT01	5930511	122	61,7	11	15
Pseudomonas putida	IAC-RBcr5	LWMA01	5760798	26	62,4	9	21
Pseudomonas putida	Idaho	AGFJ01	6363067	839	61,6	8	22
Pseudomonas putida	INSali382	LSUZ01	6490841	246	61,6	8	22
Pseudomonas putida	IOFA1	LGRH01	5708798	188	61,4	8	23
Pseudomonas putida	JCM 18452	BBDB01	6284821	823	62	10	17
Pseudomonas putida	JCM 18798	BBDC01	6577590	924	61,6	10	16
Pseudomonas putida	JCM 9802	BBDA01	6035229	472	61,7	8	22
Pseudomonas putida	JLR11	LDJF01	6100369	39	61,6	8	22
Pseudomonas putida	JQ581	LWDW01	6563906	93	62	10	17
Pseudomonas putida	KB9	LVHH01	5751833	106	62,2	7	24

Pseudomonas putida	KF703	BBQL01	6434897	135	62,1	10	17
Pseudomonas putida	KG-4	AYRY01	5586914	91	63	12	20
Pseudomonas putida	KT2440	AE015451	6181863	1	61,5	8	22
Pseudomonas putida	LF54	AOUR02	5625016	180	61,3	8	23
Pseudomonas putida	LS46	ALPV02	5874759	32	61,7	8	22
Pseudomonas putida	MC4-5222	JOJW01	6858096	1152	59,7	1	38
Pseudomonas putida	M02	JFBC01	6240608	1589	62	12	19
Pseudomonas putida	MR3	ARYY01	2994716	1268	60,8	26	7
Pseudomonas putida	MT2	2675902956*	6313443	1	61,5	8	22
Pseudomonas putida	MTCC5279	AMZE01	5215788	171	62,5	27	5
Pseudomonas putida	N1R	2681812805*	5729055	1	62	8	22
Pseudomonas putida	**NBRC 14164^T**	AP013070	**6156701**	**1**	**62,3**	10	17
Pseudomonas putida	ND6	CP003588	6202452	2	61,7	8	22
Pseudomonas putida	NFIX47	FMXW01	6383414	21	60,6	3	37
Pseudomonas putida	OUS82	AZBL01	6633308	164	61,8	10	16
Pseudomonas putida	P1	MJVE01	5140103	2899	62,2	12	19
Pseudomonas putida	PA14H7	JBOP01	5878755	7	61,9	32	33
Pseudomonas putida	PC9	2521172712*	5956110	2	62,6	12	19
Pseudomonas putida	PCL1760	LIYM01	5772078	80	61,9	8	22
Pseudomonas putida	PD1	JUHC01	5997141	37	61,8	8	22
Pseudomonas putida	PSP1	MCBG01	5993057	64	63	22	1
Pseudomonas putida	PSP2	MCBH01	5959848	59	63	22	1
Pseudomonas putida	PSP3	MCBI01	5996539	77	63	22	1
Pseudomonas putida	PSP4	MCBJ01	5964102	55	63	22	1
Pseudomonas putida	S11	ALXA01	5970749	196	62,4	12	19
Pseudomonas putida	S12	ALNR01	6284656	258	61,5	8	22
Pseudomonas putida	S16	CP002870	5984790	1	62,3	12	19
Pseudomonas putida	S610	AYJQ01	4596354	69	62	6	28
Pseudomonas putida	SF1	LDPF01	5805879	292	62,5	12	19
Pseudomonas putida	SJ3	AXDX02	5596765	279	61,6	10	16

Pseudomonas putida	SJTE-1	CP015876	5551505	207	62,3	8	22
Pseudomonas putida	SQ1	JTCJ01	5320937	1623	61,6	17	13
Pseudomonas putida	T2-2	JALX01	5524229	389	62,6	7	24
Pseudomonas putida	TRO1	APBQ01	6315212	222	61,4	8	22
Pseudomonas putida	UASWS0946	JXOG01	6001296	191	63,9	36	32
Pseudomonas putida	W15Oct28	JENB01	6320510	119	62,8	10	18
Pseudomonas putida	W619	CP000949	5774330	1	61,4	17	13
Pseudomonas putida	YKD221	BBNC01	5870708	91	61,9	8	22
Pseudomonas soli	**LMG 27941^{T}**	**FOEQ01**	**5644909**	**34**	**64**	**24**	**4**
Pseudomonas sp.	GTC 16482	BCAS01	6764392	292	61,5	8	22
Pseudomonas sp.	LAIL14HWK12_I1	2541046952*	5665646	24	62,1	11	15
Pseudomonas sp.	LAIL14HWK12_I3	2521172669*	5666577	23	62,1	11	15
Pseudomonas sp.	LAMO17WK12_I11	2521172641*	5560711	25	62,2	11	15
Pseudomonas sp.	LAMO17WK12_I12	2521172640*	5564094	27	62,2	11	15
Pseudomonas sp.	LAMO17WK12_I7	2521172645*	5564386	24	62,2	11	15
Pseudomonas sp.	LAMO17WK12_I8	2521172536*	5565480	25	62,2	11	15
Pseudomonas sp.	NBRC 111117	BCAT01	5532473	61	64,5	36	32
Pseudomonas sp.	NBRC 111118	BCAU01	6400771	206	61	8	22
Pseudomonas sp.	NBRC 111119	BCAV01	5415012	106	62,3	21	25
Pseudomonas sp.	NBRC 111121	BCAX01	6284301	131	61,5	8	22
Pseudomonas sp.	NBRC 111123	BCAZ01	5672465	108	62,9	13	9
Pseudomonas sp.	NBRC 111124	BCBA01	6089375	109	62,4	14	10
Pseudomonas sp.	NBRC 111125	BCBB01	6340580	150	61,6	8	22
Pseudomonas sp.	NBRC 111127	BCBD01	5785197	352	62,5	7	24
Pseudomonas sp.	NBRC 111128	BCBE01	5746848	197	62,2	7	24
Pseudomonas sp.	NBRC 111129	BCBF01	5688102	233	62,1	7	24
Pseudomonas sp.	NBRC 111130	BCBG01	5718736	118	62,8	15	11
Pseudomonas sp.	NBRC 111131	BCBH01	6192173	106	61,8	18	14
Pseudomonas sp.	NBRC 111132	BCBI01	6091541	134	61,9	9	21
Pseudomonas sp.	NBRC 111133	BCBJ01	6466925	188	61,4	11	15
Pseudomonas sp.	NBRC 111134	BCBK01	6142670	147	62	10	16

Pseudomonas sp.	NBRC 111136	BCBM01	6551757	202	61,5	8	22
Pseudomonas sp.	NBRC 111140	BCBQ01	6303060	206	62,1	9	21
Pseudomonas sp.	NBRC 111142	BCBS01	6325459	329	62,8	22	1
Pseudomonas sp.	NBRC 111143	BCBT01	6327194	125	62,8	22	1
Pseudomonas sp.	NBRC 111144	BCBU01	5429876	187	62,4	7	24
Pseudomonas sp.	P482	NZ_JHTS01	5623997	69	62,4	33	34
Pseudomonas sp.	RV120224_01c	2545824562*	6037951	40	62,4	11	15
Pseudomonas sp.	RV12022401b	2545824563*	6042872	38	62,4	11	15
Pseudomonas sp.	URIL14HWK12_I5	2518645599*	5723734	24	62,1	11	15
Pseudomonas sp.	URIL14HWK12_I8	2545555814*	5822697	27	62	11	15
Pseudomonas taiwanensis	**DSM 21245^T**	**AUEC01**	**5415134**	**67**	**61,8**	**20**	**8**
Pseudomonas taiwanensis	SJ9	AXUP01	6253055	736	61,8	12	19
Pseudomonas taiwanensis	VLB120	2558309062*	5966222	2	61,6	20	8
Pseudomonas vranovensis	**DSM 16006^T**	AUED01	5697807	36	61,5	**34**	**35**
Pseudomonas aeruginosa	**DSM 50071^T**	**NZ_CP012001**	**6291043**	**30**	**66,6**	outgroup	

* Genome accession numbers obtained from the Joint Genome Institute

Table S2. Major protein profiles of *P. monteilii* DSM 14164^T, *P. mosselii* DSM 17497^T, *P. plecoglossicida* DSM 15088^T, *P. taiwanensis* DSM 21245^T and *P. vranovensis* DSM 16006^T (m/z: mass to charge ratio; + and -: presence or absence of the corresponding protein).

m/z	*P. vranovensis*	*P. taiwanensis*	*P. plecoglossicida*	*P. mossellii*	*P. monteilli*
3011	-	-	-	-	-
3031	+	-	-	-	-
3299	-	-	-	+	-
3319	-	-	-	-	+
3327	+	+	+	-	-
3350	-	-	-	-	-
3587	+	+	+	+	-
3608	+	+	+	+	+
3649	-	-	-	-	-
4120	-	+	+	+	+
4128	+	-	-	-	-
4435	+	+	+	-	+
4449	-	-	-	+	-
4496	-	-	-	-	+
4551	+	-	-	-	-
4558	-	-	-	-	+
4809	-	-	-	+	-
4819	+	-	-	-	-
4829	+	-	-	-	-
4906	+	-	-	-	-
4950	-	-	-	+	-
4955	+	-	-	-	-
4957	-	+	-	-	-
4962	-	-	-	+	-
4969	-	-	+	-	+
4977	-	+	-	-	-
5096	-	+	-	-	-
5106	+	-	+	+	+
5117	-	+	-	-	-
5122	-	-	-	-	+
5124	+	+	+	-	-
5139	-	+	+	+	+
5153	-	+	+	-	-
5167	-	+	-	-	-
5188	-	-	-	-	-
5202	-	-	-	-	+
5264	-	-	-	-	+
5645	-	+	-	-	-
5990	-	+	+	+	+
6023	-	-	-	-	-
6054	-	-	-	-	+
6060	+	-	-	-	-
6086	-	-	-	-	-
6307	-	-	-	+	-
6321	+	-	-	-	+
6335	-	+	-	-	-
6595	-	-	-	+	-
6638	+	-	-	-	+
6653	+	+	+	-	-
6699	-	-	-	-	-
6702	-	-	-	-	+
7173	+	+	+	+	+
7213	+	+	+	+	+
7234	-	-	-	-	+
7277	-	-	-	-	+
7581	+	-	-	-	+
7606	-	+	-	-	-
7621	-	-	-	-	+
7853	-	-	-	-	-

**8240**		+	+	+	+
**8256**	+	-	-	-	-
**9102**	+	-	-	-	-
**9815**	+	-	-	-	-
**9912**	+	-	-	-	-
**9939**	-	-	-	-	+
**9957**	-	+	-	-	-
**10207**	+	-	-	-	-

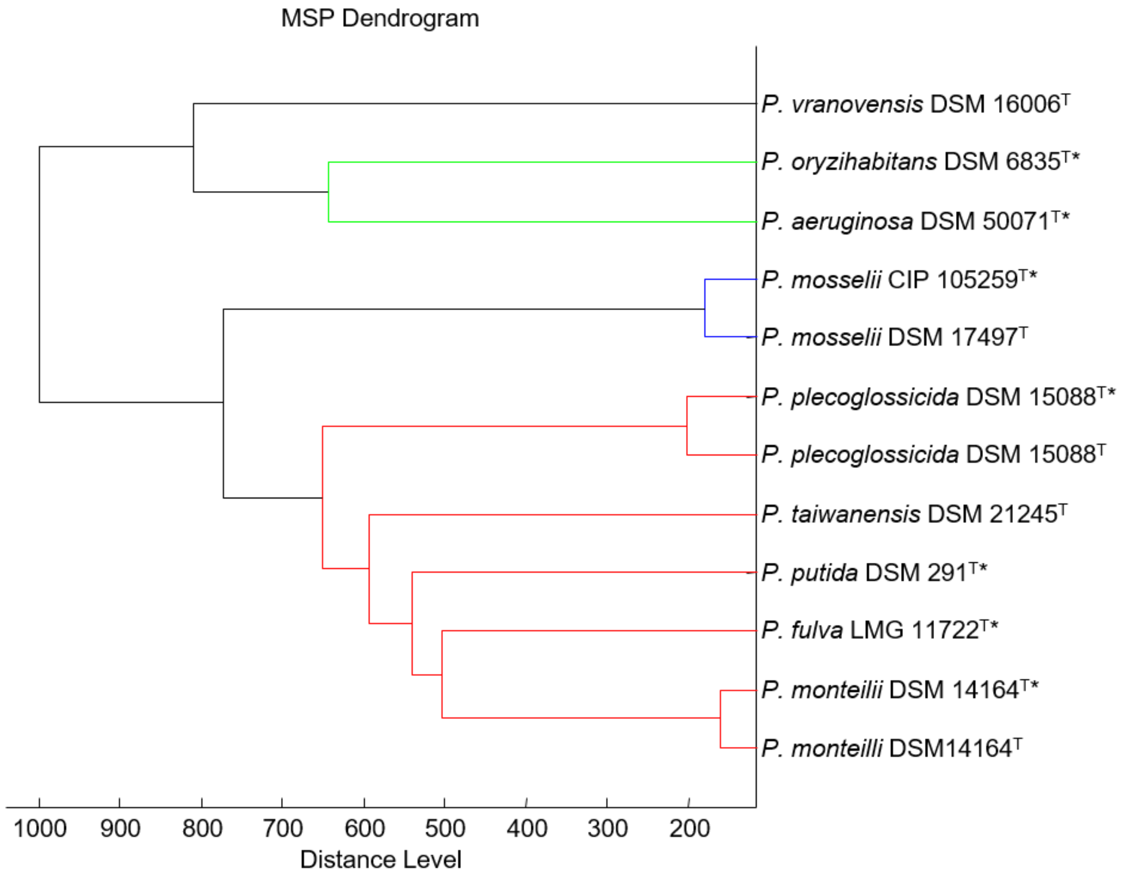

Figure S1. Dendrogram based on the comparison with the Biotyper program of the major proteins detected. The profiles of closely related type strains present in the Biotyper database are also included as a control and are labeled with an asterisk. Distances generated are arbitrary.
